# Primary liposarcoma of the ascending colon: a rare case of mixed type presenting as hemoperitoneum combined with other type of retroperitoneal liposarcoma

**DOI:** 10.1186/1471-2407-10-239

**Published:** 2010-05-27

**Authors:** Yoon Young Choi, Yong Jin Kim, So Young Jin

**Affiliations:** 1 Department of Surgery, Soonchunhyang University College of Medicine, Seoul, Korea; 2 Department of Pathology, Soonchunhyang University College of Medicine, Seoul, Korea

## Abstract

**Background:**

Liposarcoma occurs most commonly in the extremities and retroperitoneum, however, it has been rarely observed in the colon.

**Case Presentation:**

A case is reported a 41-year-old man with liposarcoma of ascending colon which was presented as hemoperitoneum and combined with a different histological type of retroperitoneal liposarcoma. He visited hospital with right lower abdominal pain and palpable mass. Laboratory data including tumor markers were within normal limits, and computed tomography revealed a 15 × 10 cm sized enhancing soft mass. Right hemicolectomy was performed, and after that, a further large retroperitoneal mass was revealed and this was also radically excised. Mixed-type colon liposarcoma and well differentiated type of retroperitoneal liposarcoma were diagnosed in pathologic report. The patient has remained free of disease for 24 months.

**Conclusions:**

No standardized guidelines have been established for its treatment because too small a number of cases have been reported, but surgical resection was considered the treatment of choice.

## Background

Liposarcoma is a common sarcoma of the soft tissues in adults, and it occurs most commonly in the extremities and retroperitoneum[1]. However, it has been observed rarely in the gastrointestinal system, and colon liposarcoma is extremely uncommon. We report a new case of ascending colon liposarcoma, and to the best of our knowledge, this is the first case of mixed-type colon liposarcoma presenting as hemoperitoneum, and the first case of combined with liposarcoma in colon and retroperitoneum.

## Case Presentation

### Clinical features

**Figure 1:**
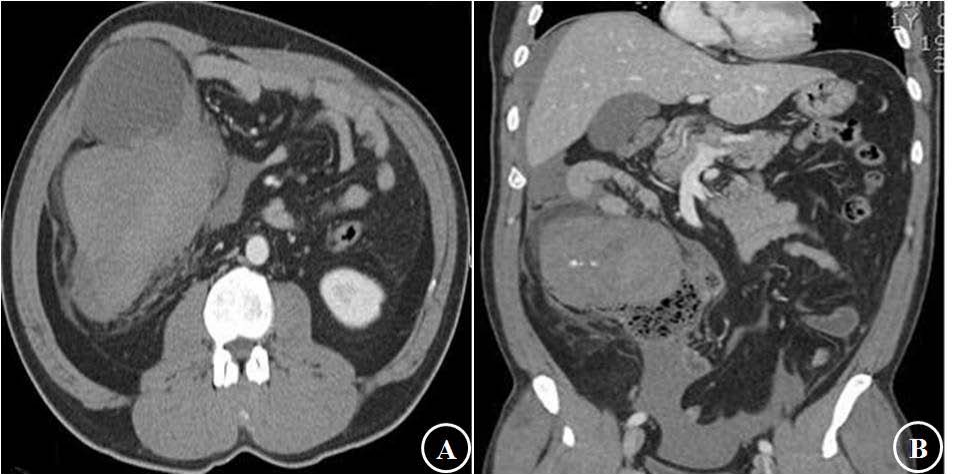
**Preoperative computed tomography(CT) findings**. A: a 15 × 10 cm sized solid mass with cystic lesion which was located in ascending colon, B: the mass was slightly enhanced and some of high density fluid collections in pelvic cavity.

A 41-year-old man visited the emergency room with abdominal pain and a palpable abdominal mass for 1 day. He had no history of disease apart from an appendectomy performed 1 year ago, and histological examination of the appendix showed features of appendicitis only. A large, soft, tender mass was palpable in the right lower abdomen. Laboratory data upon admission were within normal limits, and tumor markers such as carbohydrate antigen (CA) 19-9, carcinoembryonic antigen (CEA), and alpha-fetoprotein (AFP) were also normal. A few hours later, he complained of severe abdominal pain, and the abdominal mass was enlarged. Computed tomography (CT) revealed a 15 × 10 cm slightly enhancing solid mass, with a cystic lesion that was located in the ascending colon, without any enlarged lymph nodes. Some high-density fluid collections were observed in the pelvic cavity (Figure 1). The hemoglobin level was rechecked and it was found to have decreased from 13.1 to 12.0 g/dl.

An emergency laparotomy with midline incision was performed. About 800 ml of blood was in the pelvic cavity and a huge, lobulated, oozing soft mass that had ruptured was noted in the ascending colon. The patient underwent a right hemicolectomy. After the colon was excised, a further large retroperitoneal mass (measuring about 10 cm in the largest dimension) was revealed and this was also radically excised.

**Figure 2:**
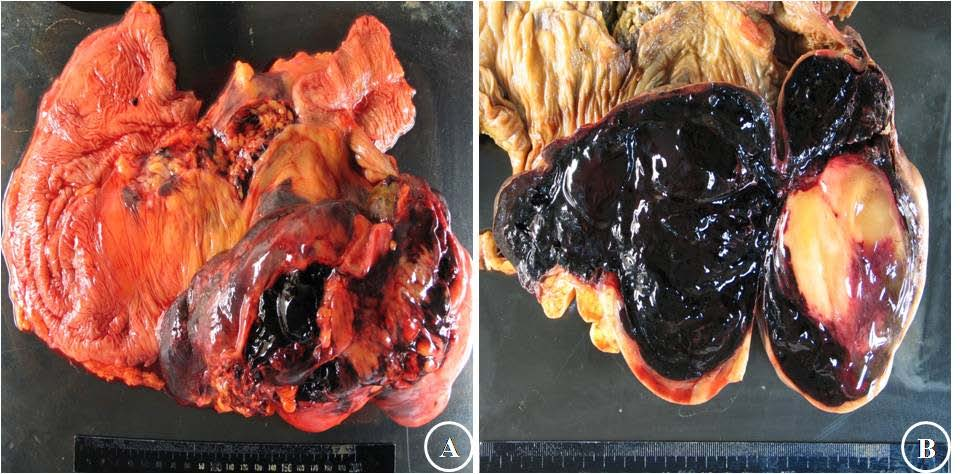
**Macroscopic findings of the colon mass**. A huge lobulated soft mass, measuring 20 × 13 × 10 cm, is noted at the ascending colon(A). Cut surface is mostly hemorrhagic gelatinous with small portion of yellowish gray solid nodule(B).

**Figure 3:**
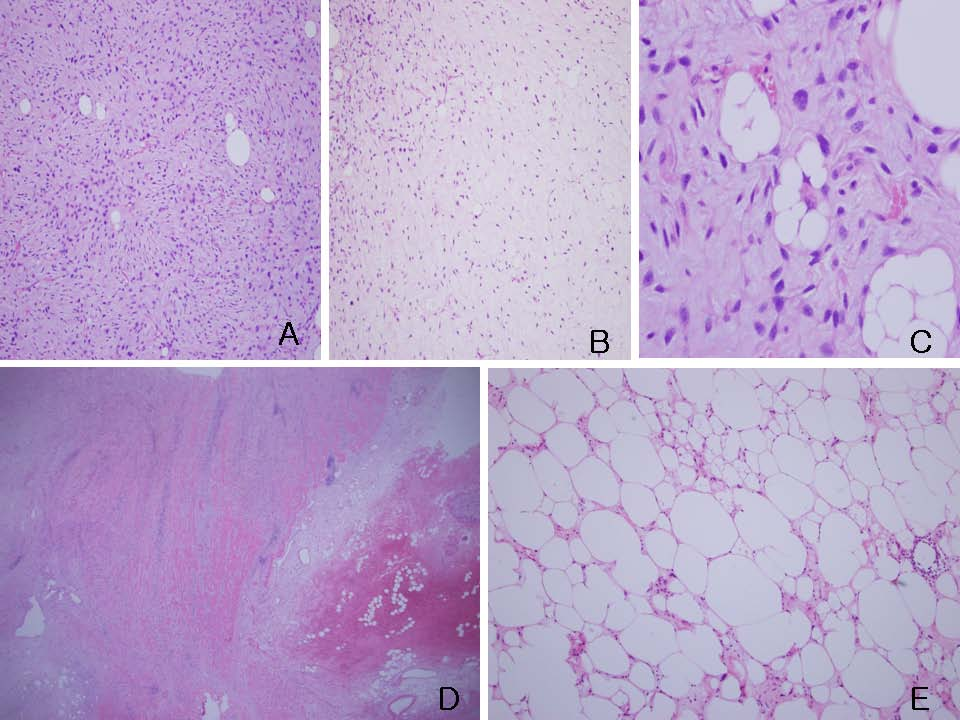
**Microscopic findings of the colon mass and retroperitoneal masses**. Proliferation of atypical spindle or stellate cells(A) focally supported by rich arborizing vasculature on the background of rich ground substance(B) with rare lipoblasts(C), tumor cells extend from the subserosa upward to the submucosa(D), most of tumor consists of mature fat cell-like cells and a few atypical spindle cells or lipoblasts(E). (A, B & E: H-E, ×100, C: H-E, ×400, D: H-E, ×12.5).

Macroscopic examination of the ascending colon revealed a large, lobulated, dumbbell-shaped soft mass that measured 20 × 13 × 10 cm, along the mesenteric border (Figure 2A). A portion of tumor was exposed and underlying hematoma was observed. Upon opening the intestine, the mucosa was discolored but intact. Upon sectioning, the cut surface was soft, friable, dark-red, and gelatinous, with a focal, yellowish-gray, solid portion, and hemorrhage (Figure 2B). Microscopic examination demonstrated loose proliferation of stellate or spindle cells on myxoid stroma, with occasional mature lipocyte-like cells (Figure 3A). They were supported by thin arborizing vasculature (Figure 3B). Rare multivacuolated lipoblasts were found (Figure 3C). Tumor cells extended upward to the submucosa, accompanied by extensive submucosal hemorrhage, but the overlying mucosa was intact (Figure 3D). The retroperitoneal masses were multiple, isolated, or conglomerated, golden to pale-yellow, glistening solid nodules, which measured 12 cm in their largest diameter. Their microscopic findings were compatible with well differentiated liposarcoma (Figure 3E). Immunohistochemical staining was negative for CD117 and positive for S-100 protein.

After surgery, the patient was discharged without any complications, and has been well, without any evidence of recurrence or metastasis for 24 months with outpatient follow up every 6 months.

## Conclusions

Liposarcoma is one of the most common soft tissue sarcomas, and represents 20% of mesenchymal malignancies. It tends to occur in the retroperitoneum and deep soft tissues of the trunk and extremities in adults[2,3]. It is unlike lipoma and relatively rare in the fat-rich areas such as the subcutaneous tissue, intestinal tract, and mesocolon[3]. Historically, liposarcoma has been divided into five subtypes according to the World Health Organization: well differentiated, dedifferentiated, myxoid, pleomorphic, and mixed type[4].

Liposarcomas rarely involve the gastrointestinal tract[5-16], and a primary liposarcoma of the colon is extremely uncommon. A recent textbook on gastrointestinal pathology cites no cases of colonic liposarcoma[17], and to the best of our knowledge, only nine cases of primary liposarcoma of the colon have been reported worldwide[5-13]. However, mixed-type primary liposarcoma of the colon has never been reported. Mixed-type liposarcoma is defined as a tumor that demonstrates the morphological features of myxoid, pleomorphic, and/or well differentiated/dedifferentiated liposarcoma[4]. This subset accounts for approximately 4% of all liposarcomas and mostly develops in the abdomen.

Clinical features of primary liposarcoma of colon are variable and nonspecific: abdominal pain, diarrhea, weight loss, anemia, and hematochezia, constipation and sometimes, an abdominal mass may be palpable (Table 1)[5-13]. However, no reports have described primary liposarcoma of the colon presenting as hemoperitoneum; in our case, hemoperitoneum may be caused by the necrosis which was progressed by overgrowing tumor. Most of the published cases are of large tumors that ranged in greatest dimension from 6 to 12 cm (in our case, the tumor size was 20 cm, but it might have been enlarged by internal bleeding), and these are common among liposarcomas in general[3]. Most interesting thing in present case is that other types of liposarcoma were found simultaneously in colon and retroperitoneum; the retroperitoneal liposarcoma was well differentiated type, and colonic one was mixed type. In the operative field, the retroperitoneal liposarcoma was found after removal of the colon and it appeared that the tumors were separate with no apparent contact.

**Table 1:** Review of reported cases of primary liposarcoma of the colon.

Author	Age/Sex	Presentation	Location	Tumor size (cm)	Histological subtype	Follow-up
Wood et al.	62/F	Pain and palpable mass	ICV	7.5 × 12	MX	Died 2 yrs
Parks et al.	54/F	Abdominal discomfort, diarrhea, anemia, and weight loss	AC	6 × 4	PM	NA
Magro et al.	65/F	Pain and intussusception	Cecum	5	WD	Alive 6 mo
Chen	52/F	Pain and hematochezia	DC	7.5 × 5	WD	Alive 2 yrs
Gutsu et al.	46/M	Pain and palpable mass	AC	12 × 11	MX	Alive 1 yr
Shahidzadeh et al.	56/F	Hematochezia	AC	3.5 × 2.8	WD	NA
Chaudhary et al.	66/F	Pain, hematochezia, weight loss, and palpable mass	DC	6 × 3	WD	Alive 6 mo
Jaboui et al.	69/M	Pain, constipation, and weight loss	DC	7 × 6	DD	Alive 10 mo
D'Annibale et al.	79/F	Pain, constipation, and weight loss	AC	5 × 5.2	PM	Died 2.5 yrs
Current case	41/M	Pain and palpable mass	AC	20 × 13	Mixed	Alive 2 yrs

ICV = ileocecal valves; AC = ascending colon; DC = descending colon; MX = myxoid; PM = pleomorphic; WD = well differentiated; DD = dedifferentiated; NA = not available.

Most studies have treated primary liposarcoma of the colon with complete wide excision[5-13]; in our case, right hemicolectomy was performed for complete remove of colon liposarcoma and all gross retroperitoneal mass was excised widely. A treatment protocol has not been established because of the small number of reported cases. The effects of chemotherapy for liposarcoma has not been yet unknown[18], but radiotherapy has shown to affect survival rates[19]. The patient was discussed with a medical oncologist and no adjuvant therapy was recommended but it was suggested that regular clinical follow up every 6 months was needed.

The prognosis of primary liposarcoma of the colon is not known. In the first case reported by Wood and Morgenstern[5], tumor recurrence developed 3 years after surgery for myxoid colon liposarcoma. Some factors have been accepted as indicators of poor prognosis: age >45 years, presence of round cells, necrotic areas within the mass[3], and dissemination of the disease[12]. Although our patient had necrotic areas within the mass, and the tumor could be disseminated due to ruptured hemoperitoneum, he is still alive after 24 months without any recurrence.

The present case is extremely rare because it is mixed type, presenting as hemoperitoneum and was combined with a different histological type of retroperitoneal liposarcoma. No standardized guidelines have been established for its treatment because too small a number of cases have been reported, but surgical resection was considered the treatment of choice in our case. A larger number of cases will be necessary to determine whether additional treatment such as chemotherapy and radiotherapy is necessary or effective in liposarcoma of colon.

## Consent

Written informed consent was obtained from the patient for publication of this case report and accompanying images. A copy of the written consent is available for review by the Editor-in-Chief of this journal

## Competing interests

The authors declare that they have no competing interests.

## Authors' contributions

YYC participated in data collection and conceived the design. SYJ, pathologist, assembled pathologic data and reviewed pathology. YJK is the operating surgeon, collected data and participated in critical review. All authors read and approved the final manuscript.

## Pre-publication history

The pre-publication history for this paper can be accessed here:

http://www.biomedcentral.com/1471-2407/10/239/prepub

## References

[B1] Kindblom LG Meis-Kindblom JM Enzinger FM Variants of liposarcoma Am J Surg Pathol 1995 19 605 606 author reply 606-608 10.1097/00000478-199505000-00016 7779180

[B2] Fletcher CD Unni KK Mertens F Eds World Health Organization Classification of Tumors Pathology and Genetics of Tumors of Soft Tissue and Bone 2002 Lyon, France: IARC Press 35 46

[B3] Enzinger FM Weiss SW Soft Tissue Tumors 1995 3 St Louis, MO: Mosby, Inc

[B4] Fletcher CD Unni KK Mertens F World Health Organization Classification of Tumors Pathology and Genetics of Tumors of Soft Tissue and Bone 2002 Lyon, France: IARC Press 227 232

[B5] Wood DL Morgenstern L Liposarcoma of the ileocecal valve: a case report Mt Sinai J Med 1989 56 62 64 2784186

[B6] Parks RW Mullan FJ Kamel HM Walsh MY McKelvey ST Liposarcoma of the colon Ulster Med J 1994 63 111 113 8658985 PMC2449083

[B7] Magro G Gurrera A Di Cataldo A Licata A Vasquez Well differentiated lipoma like liposarcoma of the caecum Histopathology 2000 36 378 380 10.1046/j.1365-2559.2000.0855d.x 10841651

[B8] Chen KT Liposarcoma of the colon: a case report Int J Surg Pathol 2004 12 281 285 10.1177/106689690401200312 15306943

[B9] Gutsu E Ghidirim G Gagauz I Mishin I Iakovleva I Liposarcoma of the colon: a case report and review of literature J Gastrointest Surg 2006 10 652 656 10.1016/j.gassur.2005.09.014 16773759

[B10] Chaudhary A Arora R Sharma A Aggarwal S Safaya R Sharma S Primary colonic liposarcoma causing colo-colic intusussception: a case report and review of literature J Gastrointest Cancer 2007 38 160 3 10.1007/s12029-008-9031-1 18972225

[B11] Shahidzadeh R Ponce CR Lee JR Chamberlain SM Liposarcoma in a colonic polyp: case report and review of the literature Dig Dis Sci 2007 52 3377 80 10.1007/s10620-007-9806-4 17393311

[B12] D'Annibale M Cosimelli M Covello R Stasi E Liposarcoma of the colon presenting as an endoluminal mass World J Surg Oncol 2009 7 78 10.1186/1477-7819-7-78 19852822 PMC2771004

[B13] Jarboui S Moussi A Jarraya H Ben Mna K Abdesselem MM Kourda A BenJilani S Guettier C Zaouche A Primary dedifferentiated liposarcoma of the colon: a case report Gastroenterol Clin Biol 2009 33 10-11 1016 8 10.1016/j.gcb.2008.11.014 19272723

[B14] Mohandas D Chandra RS Srinivasan V Baskar A Liposarcoma of the ileum with secondaries in the liver Am J Gastroenterol 1972 58 172 176 5056514

[B15] Mansour KA Fritz RC Jacobs DM Vellios F Pedunculated liposarcoma of the oesophagus: a first case report J Thorac Cardiovasc Surg 1983 86 47 50 6887958

[B16] Shokouh-Amiri MH Hansen CP Moesgaard F Liposarcoma of the stomach: a case report Acta Chir Scand 1986 152 389 391 3739551

[B17] Fenoglio-Preiser GM Noffsinger AE Stemmermann GN Lantz PE Listrom MB Rilke FO Gastrointestinal Pathology, An Atlas and Text 1998 2 New York, NY: Lippincott-Raven Publishers 1198 12019872652

[B18] Jones RL Fisher C Al-Muderis O Judson IR Differential sensitivity of liposarcoma subtypes to chemotherapy Eur J Cancer 2005 41 2853 2856 10.1016/j.ejca.2005.07.023 16289617

[B19] Strander H Turesson I Cavallin-Stahl E A systematic overview of radiation therapy effects in soft tissue sarcomas Acta Oncol 2003 42 516 531 10.1080/02841860310014732 14596510

